# Supplementary material for: Genetic and Methylation Analysis of CTNNB1 in Benign and Malignant Melanocytic Lesions
Source: Cancers (Basel). 2022 Aug 23;14(17):4066. doi: 10.3390/cancers14174066 (PMC9454999; doi:10.3390/cancers14174066)
Supplement: Supplementary file 1 [file cancers-14-04066-s001.zip › Supplementary Table S1.pdf]

Supplemental Table S1: Clinical characteristics from control groups used for comparison adapted after [24]

GROUP	HISTOPATHOLOGICAL DIAGNOSIS	GENDER	AGE AT DIAGNOSIS	LOCALISATION	TUMOR THICKNESS (MM)	VITAL STATUS	FOLLOW-UP (WEEKS)
**MELANOMA**	NMM	m	65	Lower extremity	4.1	alive	4.1
**MELANOMA**	NMM	f	70	Lower extremity	8.9	alive	14.1
**MELANOMA**	NMM	m	57	Torso back	6.0	alive	38.7
**MELANOMA**	NMM DD MUP	m	75	Head/neck	5.3	alive	29.3
**MELANOMA**	NMM	m	77	Lower extremity	8.4	deceased	77.1
**MELANOMA**	NMM	m	51	Head/neck	6.4	deceased	72.4
**MELANOMA**	NMM	m	69	Torso front	11	alive	156.7
**MELANOMA**	NMM	m	76	Torso back	3.4	deceased	85.0
**NEVUS**	Nevus	f	32	Torso back	na	alive	na
**NEVUS**	Nevus	m	59	Torso back	na	alive	na
**NEVUS**	Nevus	m	59	Torso back	na	alive	na
**NEVUS**	Nevus	m	29	Torso back	na	alive	na
**NEVUS**	Nevus	f	33	Torso back	na	alive	na
**NEVUS**	Nevus	m	48	Torso back	na	alive	na
**NEVUS**	Nevus	m	21	Torso back	na	alive	na
**NEVUS**	Nevus	f	22	Head/neck	na	alive	na
**SPITZ**	Spitz nevus	f	21	Torso back	na	na	na
**SPITZ**	Spitz nevus	f	19	Lower extremity	na	na	na
**SPITZ**	Spitz nevus	f	23	Lower extremity	na	na	na
**SPITZ**	Spitz nevus	m	11	Torso back	na	na	na
**SPITZ**	Spitz nevus	m	14	Head/neck	na	na	na

Abbreviations: DD, differential diagnosis; f, female; m, male; MM, millimeter; MUP, melanoma of unknown primary; na, not available; NMM, nodular malignant melanoma; PD-L1, programmed death ligand-1; unk, unknown; wt, wildtype; mut, mutated
