# Supplementary material for: Genetic and Methylation Analysis of CTNNB1 in Benign and Malignant Melanocytic Lesions
Source: Cancers (Basel). 2022 Aug 23;14(17):4066. doi: 10.3390/cancers14174066 (PMC9454999; doi:10.3390/cancers14174066)
Supplement: Supplementary file 1 [file cancers-14-04066-s001.zip › Supplementary_material.pdf]

Supplementary text

Case presentation

Case 1 (ID3): From the capillitium of a 59-year-old male patient, a melanocytic lesion was excised. Histologically, an extensive melanocytic lesion consisting of two components was seen. The covering epithelium was inconspicuous showing no melanocytes. Underlying and extending into the subcutaneous adipose tissue, nevoid melanocytes with slightly increased pigment content aggregated into large nests. On the other hand a much larger population of strongly pigment-bearing partly spindle celled, partly large epithelioid celled, melanocytes in large nests and strands were visible. To the surrounding corium and to the subcutaneous adipose tissue pistillate good demarcation (Supplemental Figure S2). Cytomorphologically isolated enlarged nuclei were present. In the nevoid part single mitoses were seen. The histological changes showed an atypical cell-rich blue nevus and molecular work up was requested to aid in making a final diagnosis. Genetics revealed mutations in *NRAS*, *hTERT* promoter, and *CTNNB1* and the diagnosis of a deep-penetrating nevus-like melanoma was favoured when viewed together with the histology synopsis. In addition, copy-number alterations observed for several genes argued against a benign process and supported recommending treatment as melanoma. A sentinel neck dissection and re-excision with safety margin was performed and showed no evidence for disease spreading. Stage IIB (after the American Joint Cancer Classification [AJCC]) was diagnosed and adjuvant therapy with low-dose interferon α was recommended. The patient favoured follow-up according to guidelines. The last follow-up showed no recurrence three months after diagnosis.

Case 2 (ID 6): A melanocytic lesion was excised from the back of a 64-year-old male patient. Histological evaluation initially diagnosed a partially spitzoid and deeply penetrating melanocytic tumor. Differentially, a malignant melanoma or an atypical spitzoid, deeply penetrating naevoid tumor was favoured and a reference report initiated. Reference favoured diagnosis of a malignant melanoma resembling a deep penetrating blue nevus with a tumor thickness of 12 mm. Staining for PD-L1 was positive in 5% of tumor cells. Additional molecular work-up was requested and showed mutations in *NF1*, the *hTERT* promoter region and *CTNNB1*, favouring diagnosis of a malignant melanoma. A sentinel lymph node extraction (SLNE) was performed showing subcapsular tumor cells resulting in stage IIIC. An adjuvant immune checkpoint inhibition with an anti-PD1-antibody was started five months after diagnosis but had to be terminated after seven months due to immune-related diabetes type I with diabetic ketoacidosis and cardio-pulmonal resuscitation after sepsis. Regular follow-up for four years have not demonstrated tumor recurrence; however, two additional melanoma and one melanoma in situ were excised.
