# Supplementary figures and images for: Genetic and Methylation Analysis of CTNNB1 in Benign and Malignant Melanocytic Lesions
Source: Cancers (Basel). 2022 Aug 23;14(17):4066. doi: 10.3390/cancers14174066 (PMC9454999; doi:10.3390/cancers14174066)

A

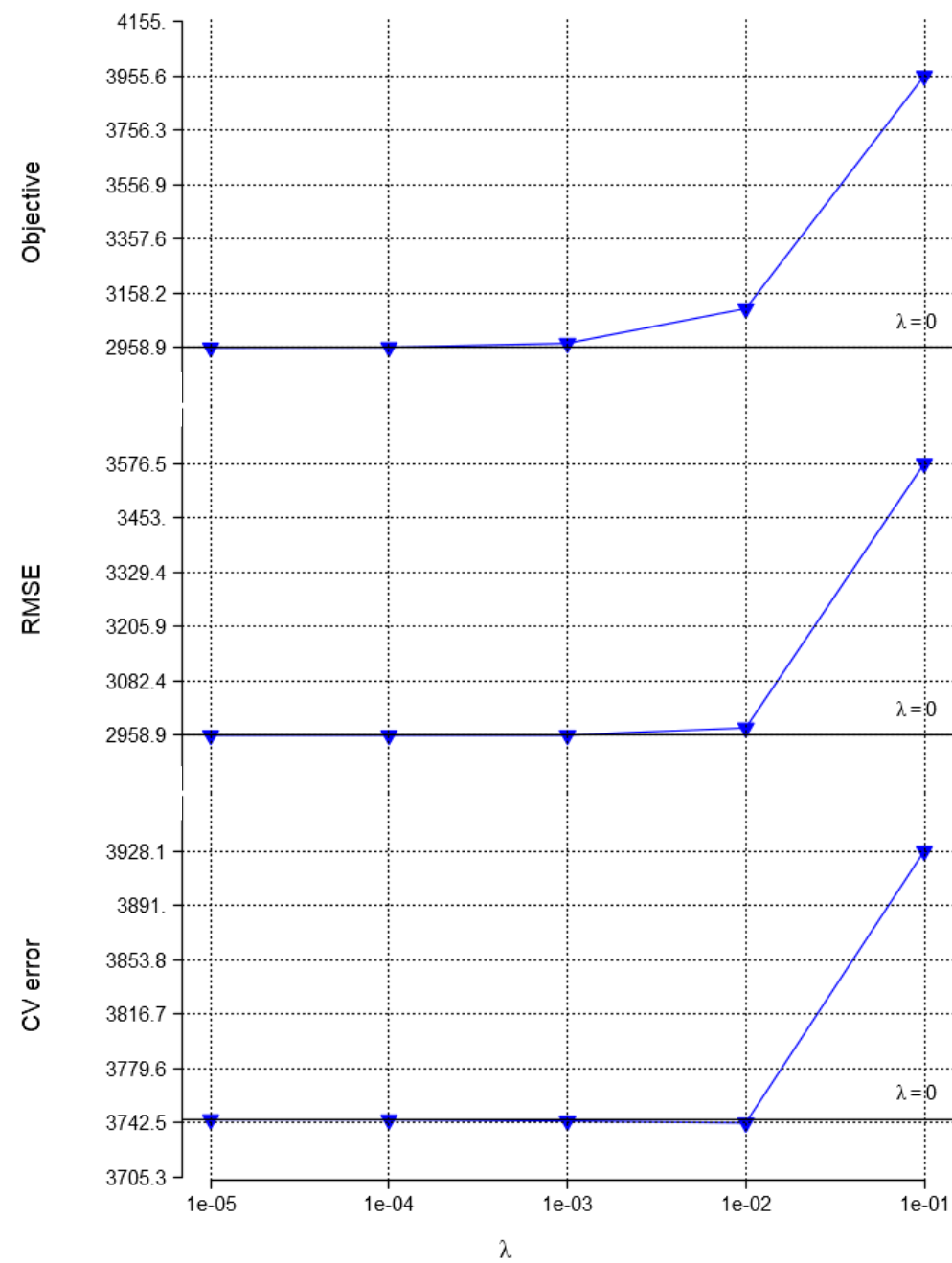

B

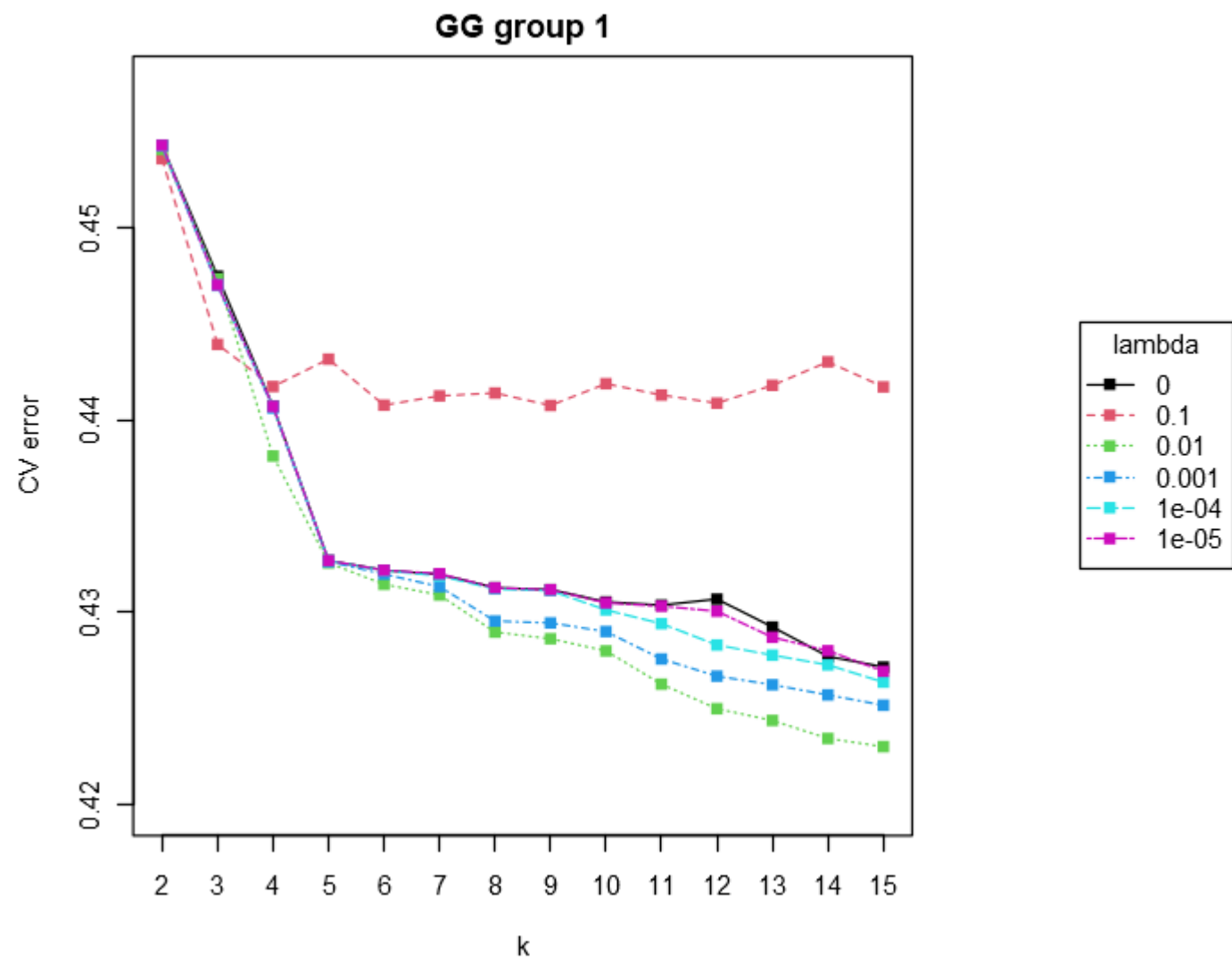

Supplement: Supplementary file 1 [file cancers-14-04066-s001.zip › Supplementary Figure S1.pdf]

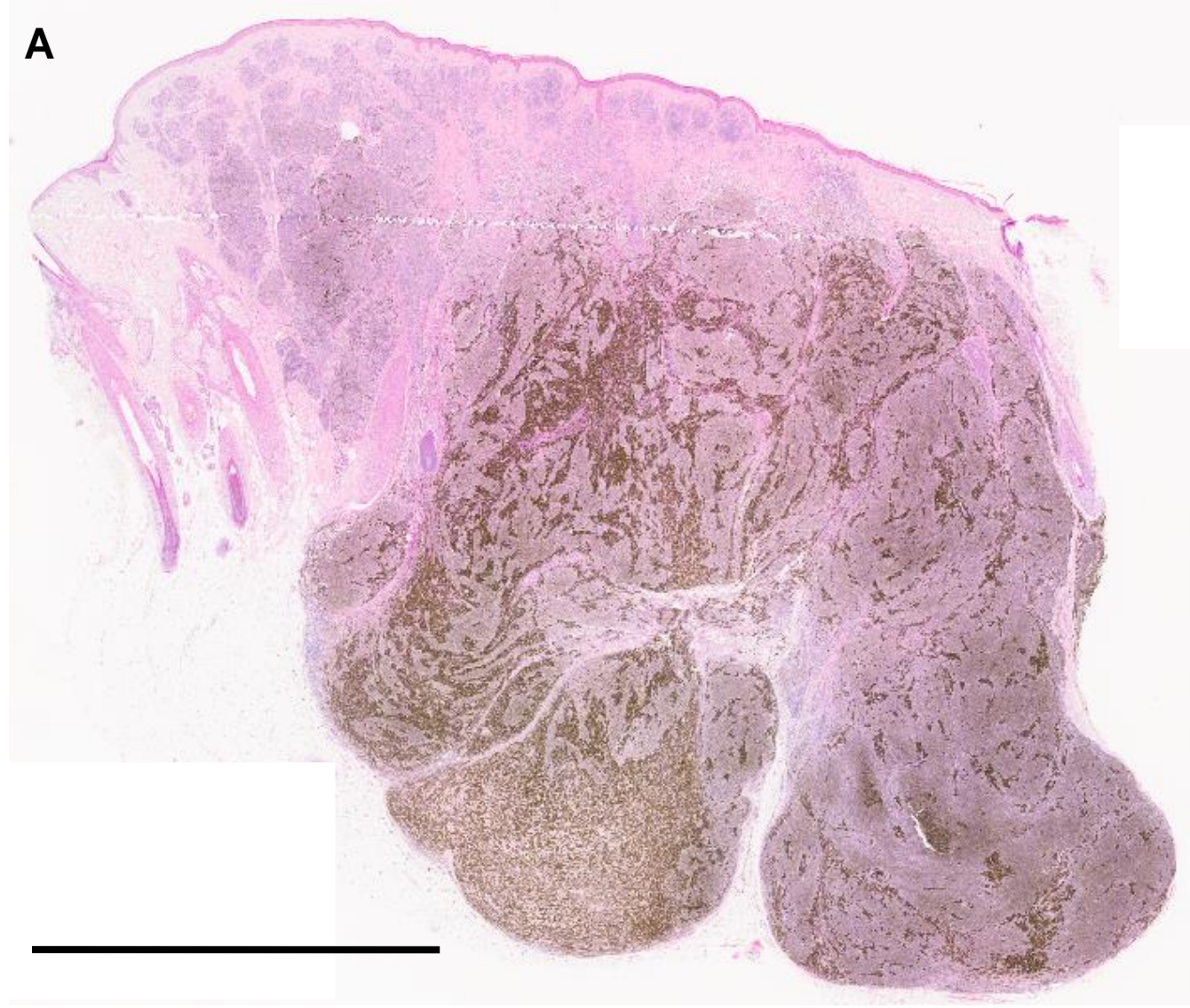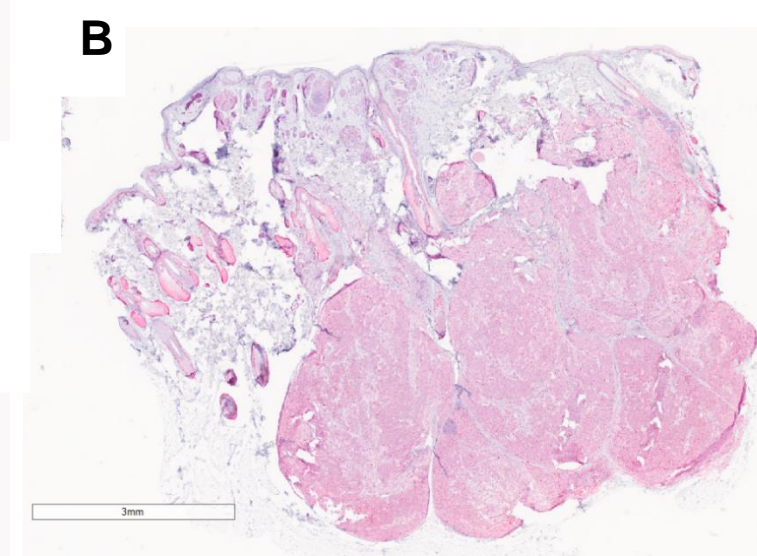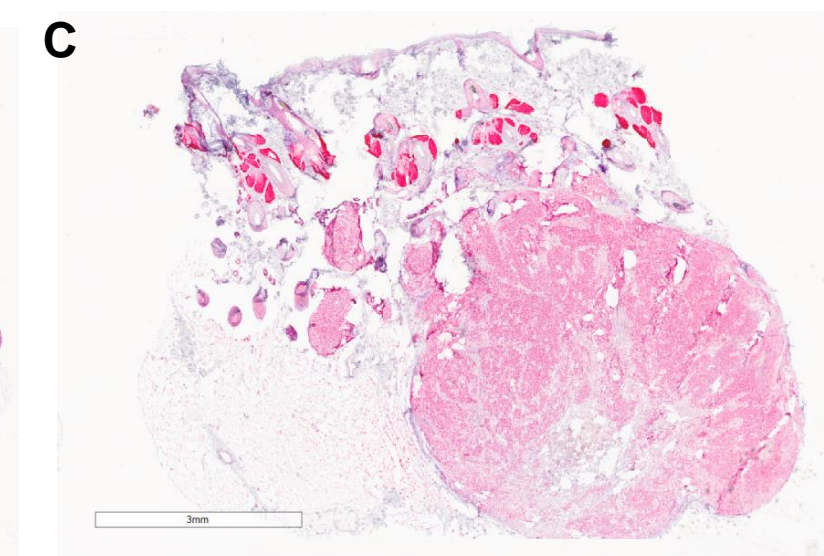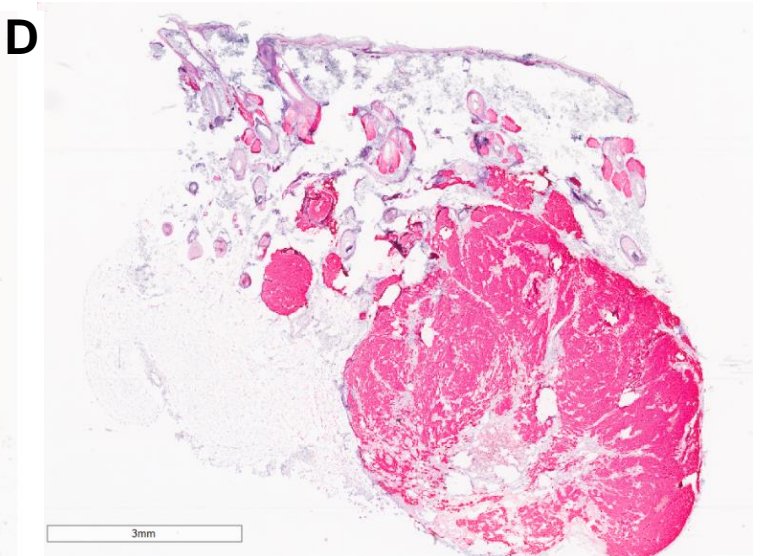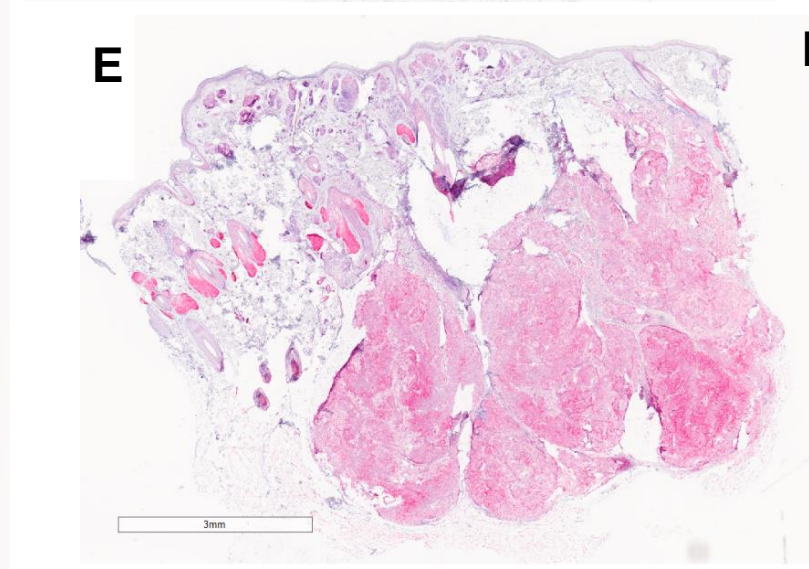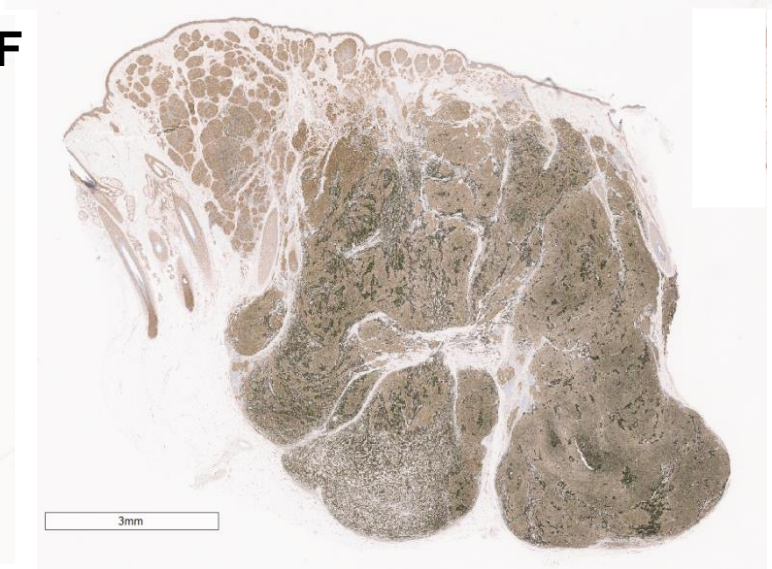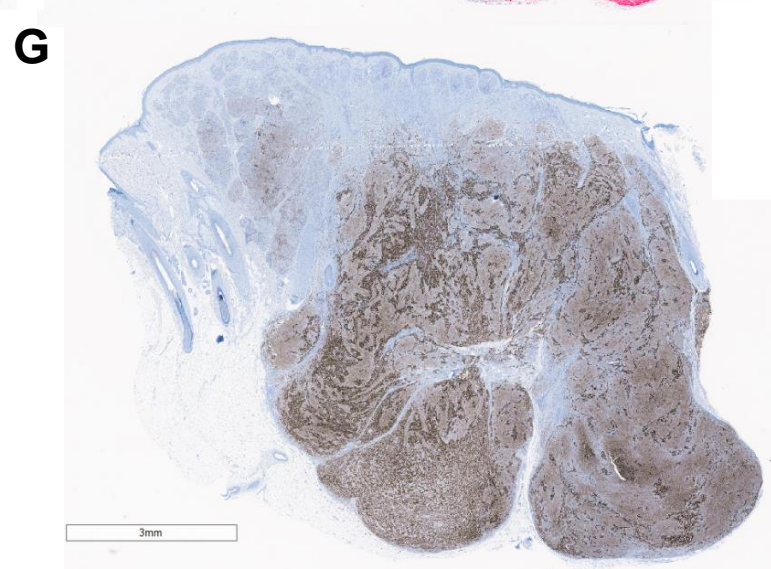

Supplement: Supplementary file 1 [file cancers-14-04066-s001.zip › Supplementary Figure S2.pdf]
